# The effects and mechanism of ginsenoside Rg1 on myocardial remodeling in an animal model of chronic thromboembolic pulmonary hypertension

**DOI:** 10.1186/2047-783X-18-16

**Published:** 2013-06-05

**Authors:** Chang-yi Li, Wang Deng, Xiu-qing Liao, Jia Deng, Yu-kun Zhang, Dao-xin Wang

**Affiliations:** 1 Department of Respiratory Medicine, the Second Affiliated Hospital of Chong Qing Medical University, 76 Linjiang Road, Yuzhong District, Chongqing 40010, China; 2 Department of Respiratory Medicine, Chongqing Fuling Central Hospital, Fuling, Chongqing 408000, China

**Keywords:** *Panax notoginseng* saponins, Ginsenoside Rg1, Chronicthromboembolic pulmonary hypertension, Myocardial remodeling, Matrix metalloproteinases

## Abstract

**Background:**

Recent studies haveshown that ginsenoside Rg1, extracted from the dry roots of *Panax notoginseng* as a traditional Asian medicine, plays an anti-fibrosis role in myocardial remodeling. However, the mechanism still remains unclear. In the present study, we investigate the effect of ginsenoside Rg1on the collagenic remodeling of myocardium in chronic thromboembolic pulmonary hypertension (CTEPH), and its potential mechanism.

**Methods:**

A rat model of CTEPH was established by injecting thrombi through the jugular vein wice in2 weeks. Four weeks later, four groups (Group A: normal rats + normal saline; Group B: normal rats + Rg1; Group C: CTEPH model + normal saline; Group D: CTEPH model + Rg1) were established. Normal saline and Rg1 were administrated by intraperitoneal injection. Ineach group, we measured the hemodynamic parameters, as well as the right ventricle to left ventricle (RV/LV) thickness ratio. Myocardial tissue sections of the RV were stained by hematoxylin-eosin +gentian violet and the morphological characteristics were observed by light microscopy. The matrix metalloproteinases (MMP) -2 and −9 were detected by the western blot.

**Results:**

Compared with Group A and Group B, the right ventricular systolic pressure was significantly increased in Group C and significantly decreased in Group D. Compared with Group A and Group B, the RV/LV thickness ratio of the rats was significantly higher in Group C and Group D. There was significant fibrosis with collagen in Group C compared with Group A and Group B, and less significant changes in Group D were observed compared with those in Group C. The expression of MMP-2 and MMP-9 exhibited a significant decrease in Group C and was also significantly decreased in Group D compared withGroup A and Group B. Also, a negative linear relationship was shown between collagen-I and the expression of MMP-2 and MMP-9.

**Conclusions:**

Our animal study showed that ginsenoside Rg1 positively affects myocardial remodeling and pulmonary hemodynamics in CTEPH. Upregulation of the expression of MMP-2 and MMP-9 could explain the beneficial effects of ginsenoside Rg1 in CTEPH.

## Background

**Figure 1:**
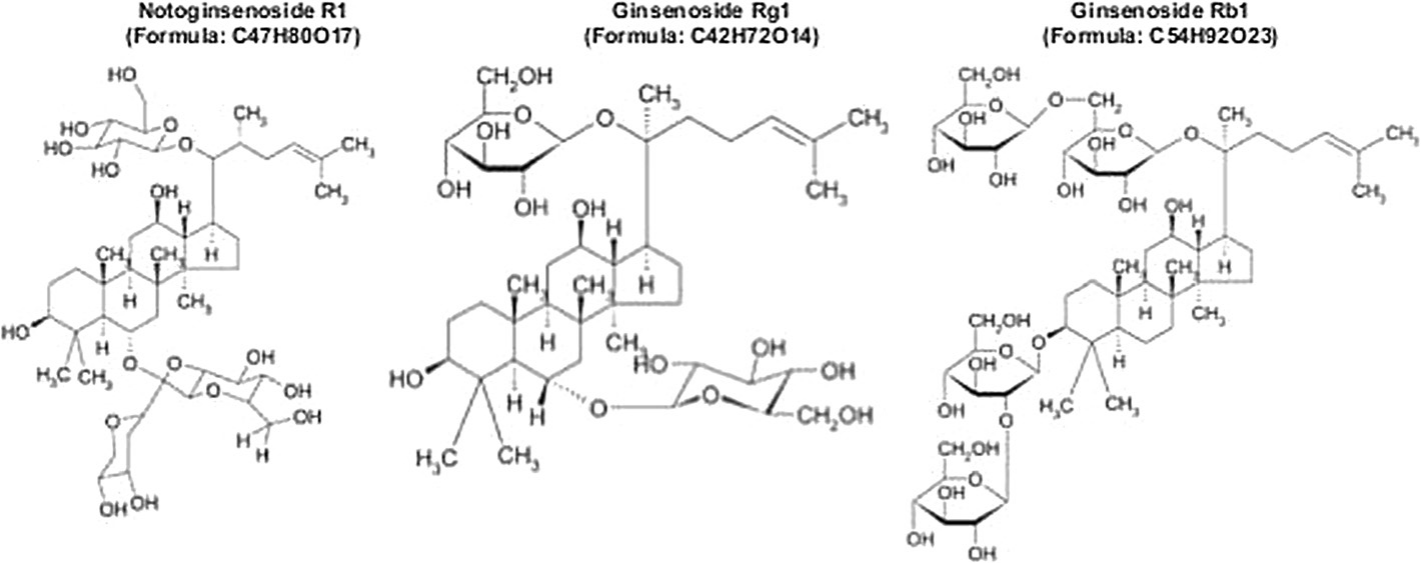
**Chemical structures of the main effective components of the total saponins of *Panax notoginseng *root.**

Pulmonary hypertension is a pathophysiologic syndrome resulting from different causes and is characterized by increases in pulmonary artery pressure and pulmonary vascular resistance [1,2]. Without treatment, this progressively deteriorating disease leads to right heart failure and eventually to death Therefore, attenuating right ventricular myocardial remodeling and delaying the deterioration of heart function should be the focus and basis of the treatment for pulmonary hypertension. The main function of the myocardial collagen network structure is to support the structure of myocardial cellsto maintain ventricular geometry and compliance. Collagen deposition, composed of type I and type III collagen, is one of the main elements of myocardial collagen network remodeling [3]. Therefore, promoting the degradation of collagen plays an important role in reducing ventricular remodeling. *Panax notoginseng* saponins (PNS), a type of saponin component, is extracted from the dry roots of *Panax notoginseng*, which has a long history of use as a remedy in traditional Asian medicine [4]. Pharmacological studies have shown that the content of PNS reached as high as 8% to 12% andis the major active component in *Panax notoginseng* roots [5]. The main effective components are ginsenoside Rg_1_, ginsenoside Rb_1_ and notoginsenoside R1 [6-8] (Figure 1). Recent studies have suggested that PNS is involved in the expansion of blood vessels, the reductionpf oxygen free radicals and the specific inhibition of vascular smooth muscle receptor-gated Ca^2+^ channels [9,10]. The study by Wu found that PNS can reduce the risk of coronary restenosis after percutaneous transluminal coronary angioplasty, an effect that was closely correlated to the deposition of an extracellular matrix (especiallyof collagen-I) during the repair of a coronary artery endothelial injury [11]. Ithas been reported that in a rat model with an infarcted myocardium, ginsenoside Rg1 significantly attenuated the development of myocardial fibrosis [12]. Ginsenoside Rg1 could also possibly treat hepatic fibrosis by decreasing the expression of tumor necrosis factor-α and reducing the secretion of phospholipase A2, as seen in a rat model with liver cirrhosis [13]. The anti-fibrosis role of ginsenoside Rg1 in myocardial remodeling still remains to be clarified.

This study aimed to investigate the changes in collagen during right ventricular remodeling and its regulatory mechanism in a rat model of chronic thromboembolic pulmonary hypertension (CTEPH) treated with ginsenoside Rg1 so that we can provide a new approach in the treatment of anti-myocardial remodeling.

## Methods

### Animal preparation

Healthy male Sprague Dawley (SD) rats, weighing 250 g to 300 g at the age of 2 months, were provided by the Experimental Animal Center of Chongqing Medical University, China. The animals were allowed to drink and eat freely, caged at 18°C to 20°C and in 65% to 70% relative humidity. All experimental procedures were conducted in accordance with the Guiding Principles for the Care and Use of Animals in Research and Teaching, approved by the Institutional Animal Care and Use Committee of Chongqing Medical University, China.

### Reagents and instruments

The experiments used rabbit anti-matrix metalloproteinase (MMP)-2 polyclonal antibodies (Sigma, USA), anti-MMP-9 polyclonal antibodies (Santa Cruz, USA), DAB reagent (Boster Company, China), gentian violet (GV) dye (Department of Histology and Embryology, Chongqing Medical University, China), protein lysate (Beijing Zhongshan Golden Bridge Biological, China), goat anti-rabbit IgG-HRP (Sigma,USA), mouse anti-β-actin monoclonal antibodies (Santa Cruz, USA), ginsenoside Rg1 (Chinese National Institute for the Control of Pharmaceutical and Biological Products, China), an optical imaging system (Olympus, Japan), a medical pathology image processing system (Axioskop40,ZEISS) and a multi-biological signals lead recorder (Chengdu Optical biotechnology company, China).

### Animal model

From a total of 32 SD rats, 16 were used to establish the rat model of CTEPH. The preparationof the CTEPH rat model was modified from that described before [14]. The day before embolization, a sample of 0.2 ml blood was collected from the tail vein and placed in a sterile tube under a 37°C water bath overnight. A thrombus was placed in sterilized petri dishes and divided into 3 mm × 1 mm emboli. Rats were injected intraperitoneally with 10% chloral hydrate (0.3 gkg^-1^) for anesthesia. The rats were fixed on the operating table and their right external jugular vein was exposed after a neck incision. The rats were injected with a mixture of about 15 thrombotic emboli in 2 ml saline injected at a speed of 0.5 ml.min^-1^. The rats showed symptoms of cyanosis and shortness of breath after injection. The needle was pulled out after injection and the neck was sutured after hemostasis. The CTEPH rat model was determined by chest radiographs. The experiment was repeated twice in 2 weeks in the same way for a total of 4 weeks. Anti-fibrinolytic tranexamic acid (12.5 mg.kg^-1^.d^-1^) was injected into the whole peritoneum. Besides the 32 SD rats, an additional two rats were killed in the pre-experiment to identify the anesthetic dose of chloral hydrate used in our study.

### Grouping and administration

Sixteen normal rats (8 in Group A and 8 in Group B) and 16 CTEPH model rats (8 in Group C and 8 in Group D) were given intraperitoneal injections of 0.9% saline (2 ml.d^-1^) or PNS (100 mg.kg^-1^.d^-1^). The groups were: Group A: normal rats + 0.9% saline; Group B: normal rats + Rg1; Group C: CTEPH model rats + 0.9% saline; Group D: CTEPH model rats + Rg1. The injections were givenonce daily for a total of 4 weeks.

### Detection of hemodynamics in rats

The rats were injected intraperitoneally with 10% chloral hydrate with a concentration of 0.3 g.kg^-1^ and were fixed in a supine position. After4 weeks of intraperitoneal injections, the right external jugular vein was isolated and exposed after a center-right incision through the neck. A PE-50 PVC catheter was inserted into the right external jugular vein slowly at 1 cm to 2 cm from the superior vena cava, 2 cm to 3 cm from the right atrium, and 4 cm from the right ventricle. The biological signal acquisition system was connected to record the right ventricular systolic pressure according to the catheter position judged bychanges of the pressure curve waveform.

### Determination of ventricularfree-wall thickness

Animals were sacrificed by an overdose of chloral hydrate after the determination of the right ventricular pressure. The heart was removed with saline rinse. The right ventricular (RV) and left ventricular (LV) free wall were cut from the heart. Through a cross section along the mitral valve leaflets, the RV and LVfree-wall thicknesses were measured by microscope and the RV/LV free-wall thickness ratio was calculated as an indicator for right ventricular hypertrophy.

### Hematoxylin-eosinexamination of myocardial tissue

From the tip of the right ventricle, 1 cm × 0.3 cm of free myocardial tissue was taken and immersed in a 10% methanol fixture, then embedded in a paraffin section. Butterfly shaped sections of 5-mm thickness were cut and placed on glass microscope slides stained with hematoxylin and eosin (HE) for histological analysisusing the pathological image processing system.

### Gentian violetstainingof myocardial tissue

To determinethe collagen deposition in myocardial tissues, GV staining was used in the study. Myocardial tissues were first dehydrated through a graded ethanol series, dewaxed using xylene, immediately dehydrated with 95% alcohol and 100% alcohol, GV dyed for 3 min to 5 min and were mountedusing a neutral xylene resin. Images wereacquired by an optical microscope (magnification 400×) and five random fields in each section were analyzed. GV staining was determinedusing the Image Pro PLUS software.

### Western blotting

From the tip of the RV, a sample of 1 cm × 0.3 cm of free myocardial tissue was taken and cut into pieces. A protein lysis buffer was added and the samples were quantified using Coomassie brilliant blue. Then 20-μg protein samples were added to the sample buffer for sodium dodecyl sulfate polyacrylamide gel electrophoresis (SDS-PAGE), the polyvinylidene fluoride (PVDF) membranes were transferred and samples were incubated with anti-MMP-2 antibodies (1:300) and anti-MMP-9 antibodies (1:500) at 4°C overnight. The membranes were incubated with anti-rabbit HRP-IgG antibodies (1:2,000) at room temperature for 1 h and were colored by diaminobenzidine (DAB) after the membrane had been washed repeatedly. Fluorescence band integrated optical density values were collected and the intensity of the bandswas readusing the relative integral optical density. Western blot images were analyzed by Bio-Rad’s Quantitive One software and corrected using mouse anti-β-actin monoclonal antibodies.

### Statistical analysis

Measurement dataare presented as mean ± standard error of the mean. Differences between groups were analyzed by one-way ANOVA. A level of *P* < 0.05 was considered statistically significant. Statistical analysis was done using the SPSSl0.0 statistical software.

## Results

### Effect of ginsenoside Rg1 on hemodynamics and RV/LV ratio in rats

**Figure 2:**
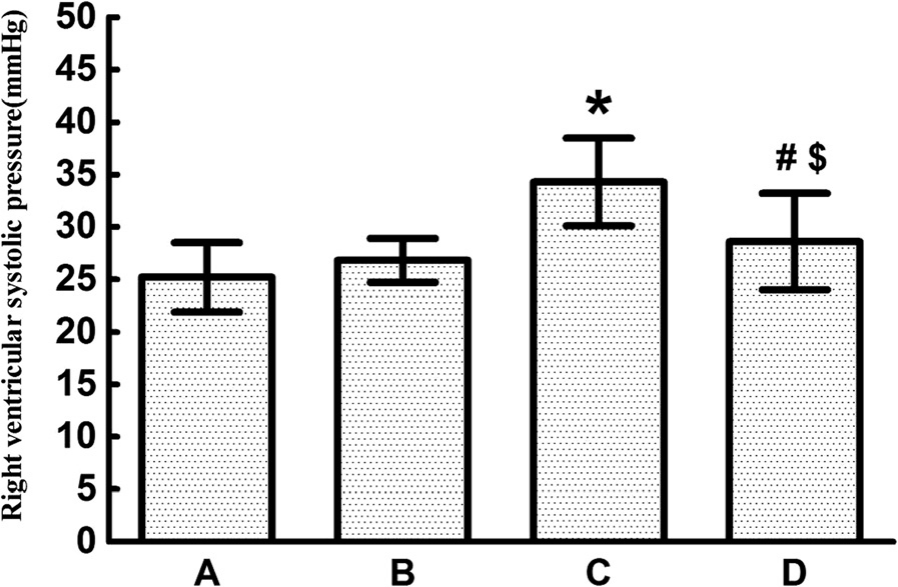
**Hemodynamics for the four different groups of rats (*n *= 8 per group).** Right ventricular systolic pressure for the four groups: Group **A**: 25.2 ± 3.3 mmHg, Group **B**: 26.8 ± 2.1 mmHg, Group **C**: 34.3 ± 4.2 mmHg, Group **D**: 28.6 ± 4.6 mmHg. Data are expressed as means ± standard error of mean. * *P* < 0.001 vs Group A and Group B; # *P* < 0.05 vs Group C; $ *P* < 0.05 vs Group A and Group B.

Compared with rats in Group A (25.2 ± 3.3 mmHg) and Group B (26.8 ± 2.1 mmHg), CTEPH rats in Group C had a significantly higher right ventricular systolic pressure (34.3 ± 4.2 mmHg). The right ventricular systolic pressure significantly decreased after ginsenoside Rg1 treatment in Group D (28.6 ± 4.6 mmHg) compared with rats in Group C. However, the right ventricular systolic pressure in Group D was still significantly higher than that in Group A and Group B (Figure 2).

**Figure 3:**
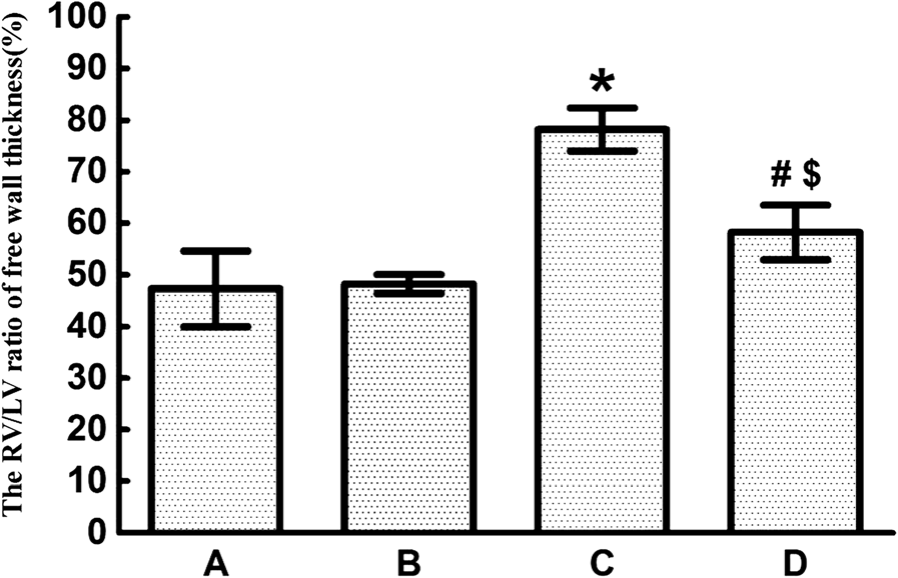
**RV/LV ratiofor the four different groups of rats (*n *= 8 per group).** RV/LV ratio of free-wall thicknessfor the four groups: Group **A**: 47.3 ± 7.3%, Group **B**: 48.2 ± 1.8%, Group **C**: 78.2 ± 4.2%, Group **D**: 58.2 ± 5.3%. Data are expressed as means ± standard error of the mean. * *P* < 0.001 vs Group A and Group B; # *P* < 0.05 vs Group C; $ *P* < 0.05 vs Group A and Group B. LV, left ventricle; RV, right ventricle.

Compared with rats in Group A (47.3 ± 7.3%) and Group B (48.2 ± 1.8%), the RV/LV thickness ratio of CTEPH rats was significantly higher in Group C (78.2 ± 4.2%). After ginsenoside Rg1 treatment, the RV/LV thickness ratioalso increased significantly in Group D (58.2 ± 5.3%), compared with Group A and Group B, but was significantly decreased compared with CTEPH rats in Group C (Figure 3).

### Histopathological examinationusing hematoxylin and eosin and gentian violet staining

**Figure 4:**
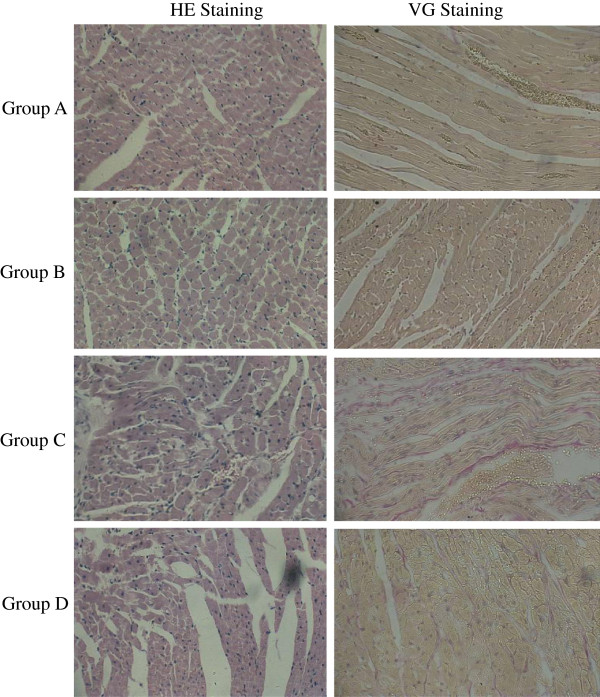
**Histopathological analysis of myocardial tissues from rats in the four groups were examined by HE and GV staining and five random fields were analyzed by microscope (400× magnification) each section (*n *= 8 per group).** GV, gentian violet; HE, hematoxylin and eosin.

The results of the histopathological analysis of the myocardial tissues from rats in the four groups are shown in Figure 4. For Group A and Group B rats, HE staining of the RV showed that the myocardial cells were arranged in neat rows, there was no inflammatory cell infiltration and the myocardial fibers were arranged in neat rowsGV staining of myocardial tissues showed that there was little pink collagen content in the myocardial fibers in Group A and Group B rats. GV staining for Group C showed that there was marked myocardial cell hypertrophy, an increased presence of disorganized partial nuclei, more inflammatory cell infiltration, disorganized muscle fibers and a large amount of pink collagen in myocardial fibers with significant fibrosisHowever, the myocardial tissues had less significant changes in Group D compared with those in Group C, suggesting alleviation of heart pathology by ginsenoside Rg1.

### Effect of ginsenoside Rg1 on the expression of MMP-2 and MMP-9 in myocardial tissue

**Figure 5:**
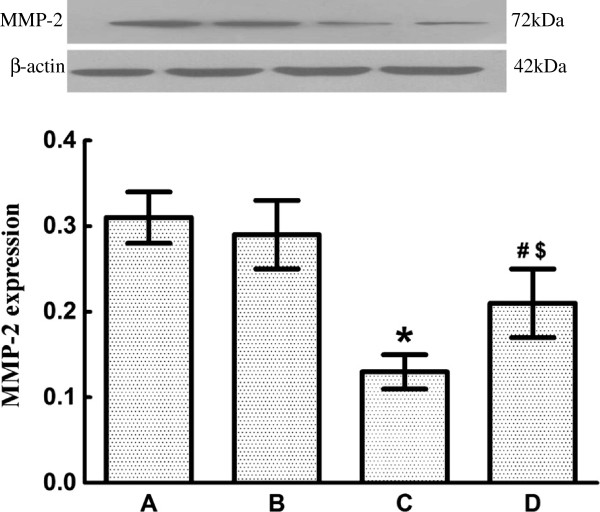
**Effect of ginsenoside Rg1 onMMP-2 expressionfor the four groups (*n *= 8 per group).** MMP-2 expression in myocardial tissuewas analyzed by western blot. Group **A**: 0.31 ± 0.03, Group **B**: 0.29 ± 0.04, Group **C**: 0.13 ± 0.02, Group **D**: 0.21 ± 0.04. Data are expressed as means ± standard error of the mean. * *P* < 0.01 vs Group A and Group B; # *P* < 0.05 vs Group C; $ *P* < 0.05 vs Group A and Group B. MMP, matrix metalloproteinase.

Compared with rats in Group A (0.31 ± 0.03) and Group B (0.29 ± 0.04), the expression of MMP-2 in CTEPH rats in Group C (0.13 ± 0.02) exhibited a significant decrease. The expression of MMP-2 also significantly decreased after ginsenoside Rg1 treatment in Group D (0.21 ±0.04) compared with rats in Group A and Group B. However, the expression of MMP-2 in Group D was still significantly higher than that in Group C (Figure 5).

**Figure 6:**
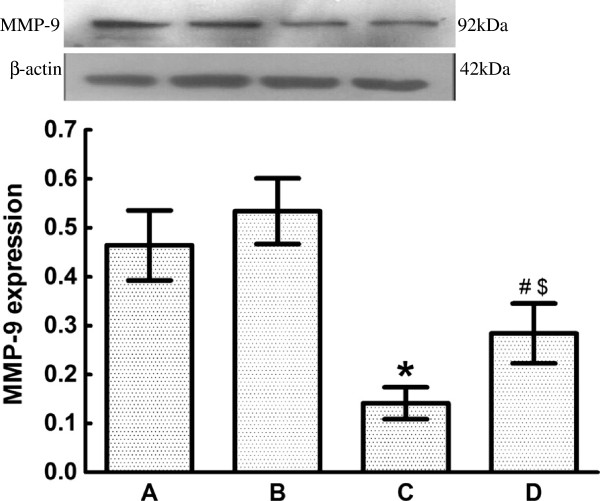
**Effect of ginsenoside Rg1 onMMP-9 expression for the four groups (*n *= 8 per group).** MMP-9 expression in myocardial tissuewas analyzed by western blot. Group **A**: 0.46 ± 0.07, Group **B**: 0.53 ± 0.06, Group **C**: 0.14 ± 0.03, Group **D**: 0.28 ± 0.06. Data are expressed as means ± standard error of the mean. * *P* < 0.01 vs Group A and Group B; # *P* < 0.05 vs Group C; $ *P* < 0.05 vs Group A and Group B. MMP, matrix metalloproteinase.

As for the expression of MMP-2, the expression of MMP-9 of CTEPH rats in Group C (0.14 ± 0.03) exhibited a significant decrease compared with rats in Group A (0.46 ± 0.07) and Group B (0.53 ± 0.06). The expression of MMP-9 also significantly decreased after ginsenoside Rg1 treatment in Group D (0.28 ±0.06) compared with rats in Group A and Group B. However, the expression of MMP-9 in Group D was still significantly higher than that in Group C (Figure 6).

### Correlationbetween the expression of MMP-2 or MMP-9 and collagen-I

**Figure 7:**
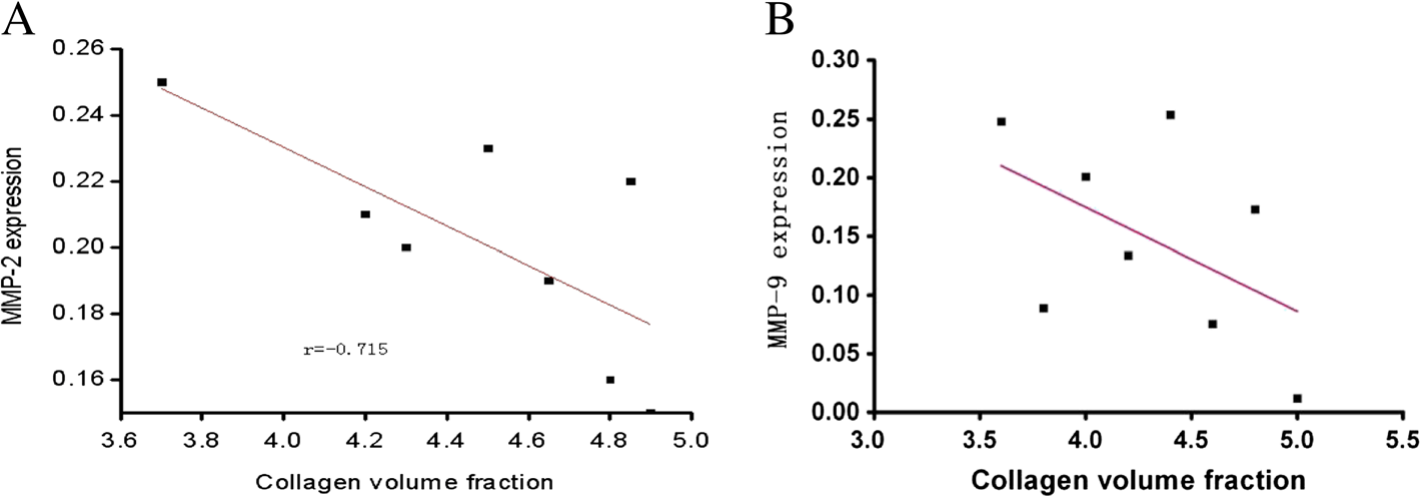
**Correlation between the protein expression of MMP-2and MMP-9with collagen-I. (A)** MMP-2 and collage-I. **(B)** MMP-9 and collage-I. Collagen volume fraction refers to the percentage of collagen-I to the whole areafrom each of the five random fieldsin each sample expressed as means ± standard error in each sample from Group D using the pathological image analysis system. Pearson’s correlation showed a negative linear relationship between the protein expression of MMP-2 and collagen-I (*r* = −0.715, *P* = 0.038)and between the protein expression of MMP-9 and collagen-I (*r* = −0.697, *P* = 0.049). MMP, matrix metalloproteinase.

In Group D, the relationship between collagen-I (expressed as the collagen volume fraction) and the expression of MMP-2 and MMP-9 was further determined using Pearson’s correlationFor the expression of MMP-2 and collagen-I, the collagen volume fractions were 3.69 ± 0.02, 4.18 ± 0.01, 4.28 ± 0.21, 4.51 ± 0.19, 4.65 ± 0.02, 4.80 ± 0.17, 4.86 ± 0.24, 4.90 ± 0.02for each sample, respectively. For the expression of MMP-9 and collagen-I, the collagen volume fractions were 3.6 ± 0.25, 3.8 ± 0.09, 4.0 ± 0.20, 4.2 ± 0.13, 4.4 ± 0.25, 4.6 ± 0.08, 4.8 ± 0.1730, 5.0 ± 0.01 for each sample respectively. Pearson’s correlation showed a negative linear relationship between the protein expression of MMP-2 and collagen-Iand between the protein expression of MMP-9 and collagen-I (Figure 7).

## Discussion

A myocardial collagen networkmainly composed of collagen-I/III, relies on the connection between myocardial structure and a direct correlation to the heart’s global systolic and diastolic function, and this was first proposed by Borg in 1981[15]. Studies have found that the deposition of collagenI/III and the imbalance of collagenI/III are the main components of myocardial collagen network remodeling [3,16]. The mechanism for impaired heart function due to interstitial fibrosis reconstruction is that myocardial collagen remodeling leads to collagen deposition, an increase in wall stiffness (decreased compliance), ventricular filling limitation, stroke volume reduction, severe interstitial fibrosis, restriction in the natural movement of myocardial cells, myocardial capillary bed reduction, residual myocardial ischemia and, ultimately, systolic and diastolic dysfunction [17]. Therefore, promoting the degradation of collagen plays an important role in the reduction of ventricular remodeling.

The major finding of the present study is that right ventricular hypertrophy, disorganized myocardial cells, myocardial cell hypertrophy, inflammatory cell infiltration, a significant increase in interstitial pink collagen and significant fibrosis in the rat heart model of CTEPH, were all improved significantly by a daily supplement of ginsenoside Rg1. That is, ginsenoside Rg1 partially reversed right ventricular remodeling in CTEPH rats. The changes in myocardial collagen were very obvious in right ventricular remodeling. Although this observation is similar to those made in a recent study, where daily administration of ginsenoside Rg1 restored morphological change of myocardial fibrosis [12], this is the first study to show the beneficial effect of ginsenoside Rg1, the use of which is based on a previous study that used an experimental model of hepatic fibrosis [13]. This study also partially reveals the mechanisms that are involved in the protective effect of ginsenoside Rg1 on attenuating myocardial remodeling.

It is believed that myocardial remodeling is to restore the structural architecture and cardiac function, which go in parallel with an increased expression and activity of MMPs [3]. MMPs are typical enzymes involved in myocardial remodeling, of which MMP-2 and MMP-9 play the mostimportant role [18]. MMP-2 and MMP-9 are gelatinases, which can degrade gelatin and normal collagen, causing the normal myocardial interstitium to be replaced by a fibrous myocardial interstitium, enlarging the ventricles and decreasing cardiac function.They haverecently aroused wide attention fortheir possible involvement in myocardial remodeling [19,20].

MMP-9has an important effect on myocardial remodeling [3,18]. Clinical studies have also found that MMP-2 activityis closely related to ventricular remodeling and heart dysfunction [21,22]. This study showed that the expression of MMP-2 and MMP-9 was low butdetectable in normal myocardial tissue, where they have an important role in the maintenance of the normal metabolism of myocardial interstitial homeostasis and the structural integrity of cardiomyocytes and the interstitium.

When the rats suffered from CTEPH and right ventricular hypertrophy, the expression of MMP-2 and MMP-9 in Group C was significantly lower than that in Group A, B and D, and was accompanied by significant myocardial collagen deposition Pearson’s correlation showed that the expression of MMP-2 and MMP-9 had a negative linear correlation with the expression of collagen-I, which is consistent with previous findings [3]. This finding indicated that decreased expression of MMP-2 and MMP-9 could be one of the mechanisms leading to collagen remodeling when rats had CTEPH and right ventricular hypertrophy. After ginsenoside Rg1 intervention, the expression of MMP-2 and MMP-9 in Group D was significantly higher than in Group C. The changein the expression of MMP-2 and MMP-9 suggested that ginsenoside Rg1 deterred right ventricular remodeling in CTEPH, which was possibly achieved by the upregulation of expression of MMP-2 and MMP-9.

In addition, the beneficial effects of ginsenoside Rg1 on myocardial remodeling need to be furtherstudied due to the small sample size. The probable side effects of the compound are unclear in thetreatment of myocardial remodeling and its mechanism still needs to be proved in future studies.

## Conclusions

In summary, our results from this study showed that daily supplementation with ginsenoside Rg1 potently attenuated right ventricular hypertrophy and myocardial remodeling in the rat model of CTEPH. Ginsenoside Rg1 also upregulated MMP-2 expression in myocardial tissue. The protective effect of ginsenoside Rg1 on myocardial remodeling may be partly associated with the upregulation of MMP-2 expression. Although our present study was performed in an experimental model, which does not directly predict a response to therapy in humans, it provides a potentially new therapeutic target for patients with CTEPHthrough ginsenoside Rg1 treatment.

## Abbreviations

CTEPH: Chronic thromboembolic pulmonary hypertension; GV: Gentian violet; HE: Hematoxylin and eosin; LV: Left ventricle; MMP: Matrix metalloproteinase; PNS: *Panax notoginseng* saponins; RV: Right ventricle; SD: Sprague Dawley; PVDF: Polyvinylidene fluoride; DAB: Diaminobenzidine; SDS-PAGE: Sodium dodecyl sulfate polyacrylamide gel electrophoresis.

## Competing interests

The authors assert that there are no conflicts of interests.

## Authors’ contributions

CL participated in the conception and design of the study. CL, WD and XL performed the animal study, HE and GV staining, cardiac histology and western blotting. JD and YZ analyzed the data and helped to draft the manuscript. DW conceived, designed and coordinated the study, and participated in the revision of the manuscript. All authors read and approved the final manuscript.
